# The Specific Characteristics in Children with Obstructive Sleep Apnea and Cor Pulmonale

**DOI:** 10.1100/2012/757283

**Published:** 2012-05-03

**Authors:** Pi-Chang Lee, Betau Hwang, Wen-Jue Soong, C. C. Laura Meng

**Affiliations:** Division of Pediatric Cardiology, Departments of Pediatrics, National Yang-Ming University and Taipei Veterans General Hospital, No. 201 Sec. 2, Shih-Pai Road, Taipei 112, Taiwan

## Abstract

*Background.* The prevalence of obstructive sleep apnea (OSA) in the pediatric population is currently estimated at 1-2% of all children. The purpose of this study was to investigate the clinical and hemodynamic characteristics in pediatric patients with cor pulmonale and OSA. *Methods.* Thirty children with the diagnosis of OSA were included. These patients consisted of 26 male and 4 female children with a mean age of 7 ± 4 years old. Five of those children were found to be associated with cor pulmonale, and 25 had OSA but without cor pulmonale. *Results.* The arousal index was much higher in children with OSA and cor pulmonale. The children with OSA and cor pulmonale had much lower mean and minimal oxygen saturation and a higher incidence of bradycardia events. All 5 patients with OSA and cor pulmonale underwent an adenotonsillectomy, and the pulmonary arterial pressure dropped significantly after the surgery. *Conclusion.* This study demonstrated that the OSA pediatric patients with cor pulmonale had the different clinical manifestations and hemodynamic characteristics from those without cor pulmonale. The adenotonsillectomy had excellent results in both the OSA pediatric patients with and without cor pulmonale.

## 1. Introduction

The prevalence of obstructive sleep apnea (OSA) in the pediatric population is currently estimated in about 1-2% of all children [1, 2]. OSA is defined as the cessation of ventilation or occurrence of significant hypoventilation during sleep, characterized by episodes of partial or complete upper airway obstruction associated with hypoxemia and/or hypercarbia. In children, OSA can occasionally result in sleep disturbances, behavioral problems, poor school performance, failure to thrive, pulmonary hypertension, and cor pulmonale [3–8]. Pulmonary hypertension and cor pulmonale can be caused by the periodic cyanosis and CO2 retention. Sdralis and Berkowitz clinically and radiologically evaluated the cardiopulmonary changes in asymptomatic adenotonsillar hypertrophy and found a significant number of children associated with alveolar hypoventilation [9]. However, information about the clinical and hemodynamic characteristics in OSA children with pulmonary hypertension and cor pulmonale is limited [10–12]. The purpose of this study was to investigate the specific clinical and hemodynamic characteristics in pediatric patients with OSA and cor pulmonale.

## 2. Methods

### 2.1. Patients

From April 1993 to March 2010, a total of 84 pediatric patients with a clinically suspected sleep disorder underwent a polysomnography (PSG) study at our institution. Thirty (36%) children with the definite diagnosis of OSA were enrolled. These patients consisted of 26 male and 4 female children with a mean age of 7 ± 4 years old (range, 2 to 14 years). In total 11 (37%) of those children were associated with an underlying disease: 4 (13%) with neurological diseases (1 with Lennox-Gastaut syndrome, 1 with Crouzen's syndrome, 1 with a Chiari malformation, and 1 with epilepsy) and 7 (23%) with obesity (5 with Prader-Willi syndrome).

Five (17%, Group I) of those children were found to be associated with cor pulmonale. The Group I patients consisted of 4 males and 1 female with a mean age of 5 ± 1 years old (range, 4 to 6 years). Twenty-five (83%, Group II) children with OSA had no cor pulmonale. The Group II patients consisted of 22 males and 3 females with a mean age of 8 ± 4 years old (range, 2 to 14 years).

### 2.2. Polysomnography

After a detailed history taking and physical examination, those children were prepared to undergo a PSG evaluation. Informed consent for undergoing the PSG was obtained from the parents of the pediatric patients before the study. All study participants underwent a full-night PSG study [13, 14], and a sleep study physician interpreted the results. No sedation or sleep deprivation was used. Most children were accompanied by a parent. The children went to bed at their usual time. The children woke up either spontaneously or were awoken in the morning to simulate the routine at home.

The following techniques were utilized to evaluate the physiological parameters of sleep: electroencephalography with bilateral central and occipital leads, electrooculography, electromyography with submental electrodes, and electrocardiography. The thoracic and abdominal muscular efforts were measured by piezoelectric sensors. The oxygen saturation was measured by pulse oximetry, and the tracheal sounds were recorded using a microphone secured to the neck. Digital videotaping with the sound recording was performed throughout the night.

Obstructive apnea was defined as a cessation of airflow through the nose and mouth for at least 2 respiratory cycles with paradoxical chest and abdominal movements. Hypopnea was defined as a reduction in the airflow through the nose and mouth with paradoxical respiratory efforts resulting in either an arousal or an oxyhemoglobin desaturation of at least 4%. The respiratory disturbance index (RDI), defined as the sum numbers of respiratory events (apneas and hypopneas) per hour of the total sleep time (TST), was used for the diagnosis. OSA was defined as an RDI greater than or equal to 5. The OSA group was sub-stratified into three groups: mild OSA (RDI: 5–19), moderate OSA (RDI: 20–39), and severe OSA (RDI: >40), respectively. The obstructive apnea index (AI) was defined as the number of obstructive and mixed apneas per hour of the TST. The arousal index was defined as the number of arousal events per hour of the TST. The desaturation index was defined as the number of desaturation events per hour of the TST. The grading scales of tonsils include (1) grade 0: tonsils fit within tonsillar fossa; (2) grade 1: tonsils < 25% of space between pillars; (3) grade 2: tonsils < 50% of space between pillars; (4) grade 3: tonsils < 75% of space between pillars; (5) grade 4: tonsils > 75% of space between pillars.

### 2.3. Echocardiography and Cardiac Catheterization

Two-dimensional and color Doppler echocardiograms were performed and recorded by a Hewlett-Packard Sonos 7500 ultrasound system with a 4 or 8 MHz transducer. Conventional parasternal short axis, parasternal long axis, apical four chamber, and subcostal views were acquired to exclude the possibility of congenital or acquired heart disease and investigate the left and right ventricular function. The severity of the pulmonary hypertension was estimated by Doppler measurements of the systolic pressure gradient between the right ventricle (RV) and right atrium (RA) with the Bernoulli equation (Δ*P* = 4*V* ^2^; pulmonary arterial pressure = 10 + Δ*P* mmHg) when tricuspid regurgitation was present.

The cardiac catheterization was performed in children with pulmonary hypertension suspected by echocardiography. After routine right- and left-sided hemodynamic recordings and angiographic studies, we calculated and evaluated (1) the pulmonary arterial pressure and resistance and (2) the systemic arterial pressure and resistance.

### 2.4. Followup Evaluation

After the detailed investigation, the patients diagnosed with moderate and severe OSA and those with OSA and cor pulmonale underwent an adenotonsillectomy procedure. Those patients were monitored for 24–72 hours in the intensive care unit and then transferred to an ordinary ward for 1 day before being discharged. Each patient came back to the clinic for followup the first week, first month, third month, sixth month, first year, and every year thereafter, respectively.

### 2.5. Statistical Analysis

All parametric data were expressed as the mean ± SD. The Students *t*-test was used to compare the demographic data and clinical and laboratory characteristics between the 2 patient groups. The proportions and rates of the incidences between the 2 groups were compared by a *z* test and Chi-square test with Yates' correction. Statistical significance was defined as a *P* value <0.05.

## 3. Results

There was no significant difference in the demographic characteristics between these 2 group patients, including the age, sex, body length, body weight, body surface area, and body mass index (Table 1). No child in Group II was associated with any underlying disease. The severity of the OSA and grading of the tonsils did not significantly differ between the Group I and Group II patients. However, the OSA children with cor pulmonale had a relatively younger age, higher body mass index, and more severe OSA, but without a statistical difference.

The result of the PSG revealed no significant difference in the RDI, AI, and desaturation index between the 2 groups (Table 1). However, the arousal index was much higher in the children with OSA and cor pulmonale (57.5 ± 65.2 versus 19.0 ± 19.7, *P* = 0.023). The children with OSA and cor pulmonale had a much lower mean and minimal oxygen saturation (87.0 ± 3.0 versus 93.9 ± 5.3%, *P* = 0.038; 42.0 ± 8.0 versus 70.5 ± 19.0%, *P* = 0.018) and a higher incidence of bradycardia events (73.0 ± 8.0 versus 11.4 ± 18.8, *P* < 0.001) during the PSG study. There was no statistical difference in the mean heart rate, minimal heart rate, and incidence of tachycardia events during the PSG study between the 2 groups.

The incidence of right ventricular hypertrophy in electrocardiography was significantly higher in children with cor pulmonale (100% versus 24%, *P* = 0.007) (Table 2). The severity of tricuspid regurgitation and pressure gradient across RV and RA were both significantly higher in children with cor pulmonale (3 ± 1 versus 1 ± 2, *P* = 0.039; 105 ± 12 versus 30 ± 16 mmHg, *P* < 0.001). Furthermore, both the systolic and diastolic systemic pressures were also statistically higher in children with cor pulmonale (135 ± 18 versus 98 ± 7 mmHg, *P* < 0.001; 88 ± 6 versus 59 ± 7 mmHg, *P* < 0.001). The systolic pulmonary pressure was significantly higher in children with cor pulmonale (112 ± 8 versus 38 ± 15 mmHg, *P* = 0.002).

Fifteen (60%) children without cor pulmonale and 5 (100%) with cor pulmonale underwent an adenotonsillectomy. In children with cor pulmonale, the pulmonary hypertension drastically decreased to a normal level after the surgery (112 ± 8 to 45 ± 13 mmHg, *P* = 0.002). A significant clinical improvement was achieved after a successful surgery in both groups.

## 4. Discussion

### 4.1. Major Findings

This study demonstrated that the OSA pediatric patients with cor pulmonale had different clinical manifestations and hemodynamic characteristics from those without cor pulmonale. The results of the adenotonsillectomy were excellent in both OSA pediatric patient groups in spite of the presence of cor pulmonale.

### 4.2. Demographic Characteristics in the Children with OSA and Cor Pulmonale

A previous report demonstrated that the risk factors for OSA included (1) age (2–7 years of age), (2) male sex (M : F = 2-3 : 1), (3) obesity (BMI ≥ 28 kg/m^2^), (4) adenotonsillar hypertrophy, and (5) craniofacial skeletal abnormalities [15]. The occurrence of OSA had been reported in children of all ages, from neonates to adolescents. In the present study, a younger age was demonstrated in the OSA children with cor pulmonale than in those without cor pulmonale which may possibly be due to (1) the relative larger tonsils and adenoids in relation to the underlying airway size and (2) a relatively poor immunity with more frequent attacks of respiratory infections.

Some previous reports demonstrated that hypoventilation and hypoxemia were exceedingly associated with obesity (body mass index of >30 kg/m^2^) [5, 16, 17]. In the present study, the mean BMI in all 5 children with OSA and cor pulmonale was larger than 30 kg/m^2^ although there was no statistical difference between the 2 groups.

### 4.3. Polysomnographic Characteristics in Children with OSA and Cor Pulmonale

A previous report demonstrated that children with a clinical diagnosis of sleep-disordered breathing may not consistently meet the PSG criteria for this disorder, and that daytime mouth breathing and habitual snoring might help clinicians recognize children who would not have sleep-disordered breathing on objective testing [18]. In the present study, both the severity of the OSA diagnosed by PSG and the grading of the tonsils could not predict the risk of pulmonary hypertension and cor pulmonale. However, the children with OSA and cor pulmonale had a statistically higher arousal index, lower minimal and mean oxygen saturation, and more frequent attacks of bradycardia events during the sleep period. This result implies that a poor quality of sleep with easy arousal may cause these OSA children to have pulmonary hypertension and cor pulmonale. Besides the fact that a long-standing low oxygen saturation can induce pulmonary vasoconstriction and frequent bradycardia episodes during sleep, it may also be related to the occurrence of pulmonary hypertension and cor pulmonale in these children.

### 4.4. Systemic Hypertension in Children with OSA

In general, systemic hypertension is less common in children than adults with OSA, and therefore would be less expected in children. However, a number of children with hypertension related to severe OSA have been reported in the literature [19, 20]. Marcus et al. [21] studied 41 children with OSA and found that 32% had a systolic and diastolic blood pressure above the 95th percentile, both while sleeping and awake. Systemic hypertension might be directly related to the severity of the obstructive apnea and level of the obesity in children. The authors attributed the rise in the blood pressure in these children to subcortical awakening, and not to hypoxemia, since there was no relation found between the pressure measurements and oximetry. This study raises the concern that children with undiagnosed OSA may develop a longstanding elevation in the blood pressure, which could result in an increased risk of cardiovascular complications later in life. In the present study, only the OSA children with pulmonary hypertension and cor pulmonale had a significantly higher systolic and diastolic systemic blood pressure, which was not directly related to the severity of the obstructive apnea and level of obesity.

### 4.5. Pulmonary Hypertension and Cor Pulmonale in Children with OSA

The early reports of childhood OSA frequently mentioned the presence of pulmonary hypertension, [22–24] resulting from recurrent nocturnal hypoxemia, hypercarbia, and respiratory acidosis. This can lead to right-sided heart failure (cor pulmonale). Left ventricular dysfunction has also been reported [19, 22, 25]. Brouillette and coworkers reported the occurrence of cor pulmonale in 55% of 22 patients with OSA [23]. Guilleminault and coworkers reported cardiac or cardiorespiratory failure in 20% of 50 patients with OSA [26]. In the present study, a lower incidence (17%) of cor pulmonale was demonstrated in our OSA children possibly due to the different ethnic characteristics and age distribution.

The relationship between pulmonary hypertension and OSA is not well demonstrated. Upper airway obstruction and chronic alveolar hypoventilation may result in an abnormal relationship between the ventilation and perfusion of the lungs. Further, hypercapnia and hypoxemia may cause respiratory acidosis and subsequent vasoconstriction of the pulmonary artery increasing the workload of the right ventricle. At the same time, the small caliper pulmonary arteries undergo remodeling and hypertrophy of the smooth muscular layers, which with time may evolve into myocardium hypertrophy, right ventricle dilation, heart failure, and cor pulmonale in some cases [12].

Wilkinson and colleagues found electrocardiogram (ECG) evidence of right heart strain in 3% of 92 children scheduled for a tonsillectomy and/or adenoidectomy; those with abnormal electrocardiograms had symptoms of OSA [27]. The present study demonstrated that 11 out of 30 OSA children (37%) had right ventricular hypertrophy on the 12-lead surface electrogram. Tal and coworkers used radionuclide ventriculography to demonstrate a reduced right ventricular ejection fraction in 37% of children with clinically diagnosed OSA, although only 7% had clinical evidence of pulmonary hypertension [25]. A previous study demonstrated that Doppler echocardiography is a safe, practical, and noninvasive method for evaluating the pulmonary arterial pressure and ventricular function in children with adenotonsillar hypertrophy [28]. We utilized transthoracic echocardiography to investigate the severity of the tricuspid regurgitation, pulmonary arterial pressure, and ventricular function in all children.

When cor pulmonale developed, it was readily reversed by treating the OSA [24, 25, 27]. In the present study, the pulmonary hypertension drastically decreased to a normal level after a successful surgery in all 5 children with cor pulmonale. Furthermore, a significant clinical improvement was achieved after a successful surgery in both OSA children with and without cor pulmonale. Postoperative pulmonary edema is a well-described complication of tonsillectomy and adenoidectomy procedures in children with OSA [29]. The mechanism is still unclear. In our patients, there was no significant evidence of postoperative pulmonary edema after the tonsillectomy and adenoidectomy.

## 5. Limitations

Because only a limited number of pediatric patients were enrolled in this study, more studies and analyses are needed to investigate the specific characteristics and risk factors in these children.

## 6. Conclusion

This study demonstrated that the OSA pediatric patients with cor pulmonale had different characteristics than those without cor pulmonale. The results of the adenotonsillectomy procedures were excellent in the OSA pediatric patients either with or without cor pulmonale.

## Figures and Tables

**Table 1:** A comparison of the demographic, clinical, and polysomnographic characteristics between the two children groups with OSA.

	Group I (*n* = 5)	Group II (*n* = 25)	*P* value
Age (y/o)	5 ± 1	8 ± 4	0.214
Sex (Male/Female)	4/1	22/3	0.913
Body Length (cm)	107.7 ± 8.6	127.0 ± 25.0	0.202
Body Weight (kg)	24.7 ± 7.1	43.4 ± 22.3	0.167
BSA (M^2^)	0.86 ± 0.15	1.24 ± 0.46	0.173
BMI (kg/M^2^)	32.8 ± 1.7	28.3 ± 6.2	0.229
Underlying Disease	0 (0%)	11 (44%)	0.332
Severity of OSAS			
Mild	0 (0%)	13 (52%)	0.274
Moderate	2 (40%)	5 (20%)	0.284
Severe	3 (60%)	7 (28%)	0.620
Grading of the tonsils	3.4 ± 0.7	3.3 ± 1.2	0.890
Polysomnography			
RDI	39.2 ± 30.8	23.4 ± 27.3	0.357
AI	10.0 ± 8.0	13.2 ± 16.0	0.739
Arousal Index	57.5 ± 65.2	19.0 ± 19.7	0.023*
Desaturation Index	51.0 ± 37.4	31.9 ± 36.8	0.404
Mean SaO_2_ (%)	87.0 ± 3.0	93.9 ± 5.3	0.038*
Minimal SaO_2_ (%)	42.0 ± 8.0	70.5 ± 19.0	0.018*
Mean HR (/min)	76.7 ± 23.6	100.4 ± 31.3	0.219
Minimal HR (/min)	44.8 ± 25.9	63.5 ± 16.3	0.087
Bradycardia events	73.0 ± 8.0	11.4 ± 18.8	<0.001*
Tachycardia events	55.0 ± 66.0	16.5 ± 26.0	0.052

Abbreviations: AI: apnea index; BMI: body mass index; BSA: body surface area; HR: heart rate; RDI: respiratory disturbance index; SaO_2_: oxygen saturation.

*: significant difference.

**Table 2:** A comparison of the electrocardiographic, echocardiographic, and hemodynamic characteristics between the two children groups.

	Group 1 (*n* = 5)	Group 2 (*n* = 25)	*P* value
Electrocardiography			
RV hypertrophy	5 (100%)	6 (24%)	0.007*
Echocardiography			
Severity of TR	3 ± 1	1 ± 2	0.039*
Pressure gradient across the RV and RA (mmHg)	105 ± 12	30 ± 16	<0.001*
Cardiac Catheterization			
Systolic SAP (mmHg)	135 ± 18	98 ± 7	<0.001*
Diastolic SAP (mmHg)	88 ± 6	59 ± 7	<0.001*
Systolic PAP (mmHg)	112 ± 8	38 ± 15	0.002*
LVEF (%)	56 ± 12	67 ± 14	0.113
Surgical intervention	5 (100%)	15 (60%)	0.225

Abbreviations: LVEF: left ventricular ejection fraction; PAP: pulmonary artery pressure; RA: right atrium; RV: right ventricle; RVEF: right ventricular ejection fraction; SAP: systemic artery pressure.

*: significant difference.
